# Community as Governor: Exploring the role of Community between Industry and Government in SLO

**DOI:** 10.1007/s00267-022-01681-0

**Published:** 2022-07-19

**Authors:** Gregory Poelzer, Rosette Frimpong, Greg Poelzer, Bram Noble

**Affiliations:** 1 Luleå University of Technology, Political Science, Luleå, Sweden; 2 School of Environment and Sustainability, University of Saskatchewan, Saskatoon, SK Canada; 3 University of Saskatchewan, Geography, Saskatoon, SK Canada

## Abstract

For many natural resource projects, the impact on Indigenous communities is a primary concern. Therefore, governance arrangements that account for the interests of companies, communities, and government are critical for the project’s success. This paper looked at two successful mining projects in northern Canada, McArthur River and Diavik, to examine the governance arrangement that led to mutually beneficial outcomes. Through an analysis of interviews and documents, we assessed both governing institutions and interactions to understand how the respective companies and communities established a high level of trust. In both cases, government took a less prominent role in the management of resources, allowing the Indigenous communities to hold a stronger role in the governance of the resources. Both Indigenous communities, therefore, built partnerships with the company around socio-economic benefits along with environmental monitoring – redefining ‘community’ in governance arrangements.

## Introduction

The governance of mineral extraction greatly affects nearby communities. Influence over the outcomes from mining operations is determined by the roles and relationships between government, companies, and communities (Prno and Slocombe 2012). Because meeting the legislative requirements is no longer a guarantee for success in mineral extraction (Prno 2013); companies must work with communities to build acceptance and approval for their operations (Thomson and Boutilier 2011; Parsons et al. 2014). This is often referred to as social license to operate (SLO). In the Canadian mining context, Indigenous communities are one of the most prominent actors involved in the governance of mineral resource development, particularly given the importance of land use, and are often conceptualized as the ‘community’ component of the government-company-community relationship. Importantly, despite all three actors bringing different expectations to a project, positive outcomes are possible when consensus and trust between parties is reached (Moffat et al. 2016) via well-defined measures (Zhang et al. 2015). Acknowledging the potential for mutual benefit, the aim of this article is to investigate the structure of governance arrangements that facilitate trust and positive outcomes in the development and management of mining operations.

Conflicting interests in land use for resource extraction, protected areas, and traditional land use have induced communities, governments, and the mining industry to look for arrangements that are satisfactory for all actors. Over the years, government has played significant roles in preventing such conflicts and ensuring that the needs of all stakeholders, especially Indigenous communities, are met in resource exploitation projects. While regulations play a major role in the acceptance of northern resource development (Poelzer 2015), obtaining a formal government license to operate and complying with regulatory requirements is no longer enough (Prno and Slocomber 2012). This has led studies on acceptance to shift to the concept of governance to understand the engagement between government, business, and civil society (Dredge and Whitford 2011; Hall 2011). These governance arrangements between state, industry, and community are essential in understanding how SLO is framed and defined (Prno and Slocombe 2012), but there is still limited work on the role of Indigenous communities in these governance arrangements. There is, therefore, a need for further work to understand how different governance models involving Indigenous actors should be understood.

Canada is known globally as a major mineral producer, due to the prevalence of deposits found throughout the country. But to position itself as a prominent mining jurisdiction, both government and companies recognized the need to build strong relationships with Indigenous communities. Indigenous communities have raised concern about profits from mining flowing South while irreversible socio-economic and environmental risks remain in the North (Bone 2012). For communities to see value in resource development, a combination of socio-economic and environmental questions must be addressed and managed in a way that sees the outcomes produce a net benefit. Benefits emerge based largely on the relationships between actors. For Indigenous communities, the result of their proximity to mining operations is mixed and a significant portion of research on the impact of mining points to problems associated with compensatory measures that may inhibit divergent voices within the community and demonstrate a failure on the part of government or companies to live up to promises (Hilson et al. 2019). Particularly because mining requires long-term land use, the need to share space between Indigenous communities and mining operations is central to the issue of preserving land for traditional activities such as hunting, fishing, trapping, and harvesting which are necessary for the vitality of Indigenous communities. However, positive relationships exist and, therefore, governance arrangements that produce an SLO warrant attention.

The adverse environmental and socio-economic impacts associated with mineral development, coupled with the need for increased stakeholder participation in mineral development decision-making processes (Prno and Sclombe 2012) have made SLO an important concept in the mining industry (Mercer-Mapstone et al. 2017). Moreover, the increasing awareness of the number of conflicts associated with the mining industries has the need for SLO in mining projects (Davis & Franks 2014). SLO refers to the acceptance and approval given by local communities to industries permitting them to operate in their territories (Thomson and Boutilier 2011). In the context of mining, “SLO is based on the idea that, in addition to government permits, mining companies also need permission or consent from the public to conduct their business” (UNDP 2018, p.94). With most mining sites in Canada located within Indigenous territory, SLO with Indigenous communities is often essential. This paper explores the governance of mining projects and the subsequent interaction that produces SLO with Indigenous communities.

To investigate these governance models, this paper engages in two tasks: 1) understanding industry-state-community relationships that exist in mining projects in northern Canada 2) examining how industry-state-community relations fall under co-management and non-co-management systems. Drawing on two cases of SLO in the mining sector in Canada – Tlicho Government and diamond mining in the Northwest Territories, under a form of a co-management government arrangement, and English River First Nation and uranium mining in Saskatchewan under a non-co-management system – this paper provides insight on the governance of resource development projects in Canada, that is transferrable to other parts of the world.

## Framing SLO in the Context of Interactive Governance Theory

### The Concept of SLO in the Mining Sector

The term ‘social license’ is attributed to a Canadian mining executive, Jim Cooney, who used it in the late 1990s to describe what he thought was a necessary condition for the successful future of the mining industry in terms of responding to society’s expectations for responsible resource development (Prno 2013; Moffat and Zhang 2014). Mining companies have been criticized because of the negative impacts of their activities on the environment. However, in response to mounting criticism, the mining industry has paid increasing attention to the environmental and social impacts of its activities, notably by embracing the concept of social license (Whitmore 2006; Thomson and Boutilier 2011). Thomson and Boutilier (2011) consider SLO from the point of view of community expectations and experiences. Therefore, obtaining SLO has become essential for extractive industries as key stakeholders are increasingly expecting the industry to contribute positively to the community in which the industry operates, and to communicate openly and engage local communities in their decision-making (Moffat and Zhang 2014). Over the last decade, there has been a global shift towards recognizing the rights of Indigenous communities regarding extractive activities on their traditional territories (Anaya 2005; Tomlinson 2019; Åhrén 2016). Considerable literature demonstrates that in most cases, government and industry continue to fail to consult with affected communities adequately and rarely achieve their consent before the exploitation of natural resources (Anaya 2004; Hanna and Vanclay 2013: Tomlinson 2019). Communities demand a greater say in the decision-making processes and the acceptance of industrial activities.

SLO is a useful and practical tool for organizations to deploy in the negotiation, implementation, and operational phases of any project. In their theoretical work on SLO, Thomson and Boutilier (2011) developed a pyramid model and flow chart that includes positions for categorizing social license. The lowest level of the pyramid is withdrawal, characterized by blockades, violence, and shutdowns. Thomson and Boutilier (2011) suggested that next levels are legitimacy and then credibility where SLO can be withheld if there is no acceptance from the communities in which an industry operates, and a company is considered to be legitimate when it has acquired the acceptance level. Approval and acceptance are often identified as key elements of social license (Thomson and Boutilier 2011; Dare et al. 2014; Gunningham et al. 2004; KPMG 2013; Lansbury and Jeannert 2015; Prno 2013; Rooney et al. 2014). Approval is when an industry is allowed to operate while acceptance is when community stakeholders have developed an understanding about industry operations and feel positively about community-industry relationships (Thomson and Boutilier 2011; Dare et al. 2014; Gunningham et al. 2004; KPMG 2013; Prno 2013; Rooney et al. 2014). Psychological identification, also known as co-ownership when viewing the pyramid from the perspective of community stakeholders, is the highest-ranking for social license, and comes after obtaining trust; this includes recognizing the values and needs of the local community and investing itself equally as a stakeholder in those goals (Thomson and Boutilier 2011). Additionally, trust is most often recognized as the underlying principle of the entire notion of social license (Thomson and Boutilier 2011; Bursey and Whiting 2015; Dare et al. 2014; KPMG 2013; Moffat and Zhang 2014; Yates and Horvath 2013).

Koivurova et al. (2015) used the SLO model to analyze how a community responded to the behaviour of eight mining companies located in Norway, Finland, Russia, and Sweden. In all cases they found legitimacy or credibility, but not trust. Prno (2013) gave an example of how a social license was successfully established for a Red Dog (zinc-lead) mine in Alaska, USA. He describes the establishment of an SLO given that community members in the region were largely in support of the mine’s operation due to its importance to the local economy. Their view was that the mine offers a fair distribution of financial benefit and ensured community members’ participation in decision-making processes (Prno 2013).

The SLO concept is particularly useful as its application works in several industries such as energy, construction, forestry, and mining (Smits et al. 2016; Melé and Armengou 2016; Edwards and Lacey 2014; Jijelava and Vanclay 2017). Most of these studies focus on the relations between industry and stakeholders involved in the SLO process. For mining, research on SLO is focused specifically on its application (Prno and Slocombe 2012), what mining companies do to obtain SLO (Fuisz-Kehrbach 2015; Prno and Slocombe 2014), and how SLO can be measured (Boutilier and Thomson 2011). Some scholars have made recommendations regarding the need to obtain SLO with communities, which include the need for early communication; transparent disclosure of information; development of conflict resolution mechanisms; and culturally appropriate decision-making (Goldstuck and Hughes 2010). In many cases this results in voluntary, formal agreements between mining companies and communities, such as Impact Benefit Agreements, where the benefit-side focuses on the opportunities brought by mining development and the impact-side seeks to address adverse socio-economic and environmental impacts. The promise is to achieve “a more sustainable mining development by…engaging in the appropriate level of consultation and providing adequate benefits and compensation” (Hitch and Fidler 2007). In these types of arrangements, we see political outcomes achieved through company-community collaboration.

While the interaction between the company and community remains key to SLO, other relationships contribute to shaping the overall outcomes. Past research points to the necessity of understanding the overarching governance of resource management and how that results in mutually beneficial outcomes, specifically the points of interaction between government, industry, and community (Lehtonen et al. 2020; Cullen-Knox et al. 2017; Prno and Slocombe 2012). This paper heeds this call to raise the level of analysis of SLO beyond company to community by looking at the broader governance arrangements.

### Governance and Mineral Exploitation

Governance can be seen as the reflection of how communities, societies, and organizations such as industries and government agencies organize themselves to make important decisions regarding the use and protection of their common resources (Armitage et al. 2018; Derkyi 2012). Some scholars perceive governance as an interactive process of steering the affairs of both state and non-state actors (Kooiman 1993). It includes the formulation and application of principles guiding those interactions and care for institutions that enable them (Kooiman et al. 2005). The transition from government to governance has yielded a broadened range of governing actors in mining projects, resulting in industry and civil society sharing governing responsibilities with governments (Ballard and Banks 2003; Lemos and Agrawal 2006; McAllister and Fitzpatrick 2010; Prno and Slocombe 2012). Government does not decide alone but needs non-governmental actors and stakeholders to contribute on issues of resource use and sustainable development.

According to Smits et al. (2016: 130), substantial involvement of stakeholders in project governance from the start of the project is crucial because SLO failures have mainly been due to a lack of stakeholder involvement in the role of governance. The concept of governance is a step towards a more dynamic field of engagement between government, civil society and industries. Their participation in the process often results in community acceptance or rejection of a project (Dredge and Whitford 2011; Hall 2011). The shift to involve stakeholders is especially relevant to the emergence of SLO as there is an increased level of demand for new input in decision-making (McMahon and Remy 2001). Moreover, the relevance of stakeholders such as community, government, and industry has increased over the years (Parmar et al. 2010); their criticism significantly affects industries’ actions (Barnett 2007), as they seek to legitimize their operations, and this requires connecting with stakeholders and meeting their needs (Chen and Roberts 2010).

The connection between governance and SLO puts the focus firmly on the interaction between actors, particularly how they navigate the institutional context to develop relationships. While Prno and Slocombe (2012) place SLO in a governance context, their framework lacks guidance as to how to analyze the quality of interactions that form the core of SLO (Thomson and Boutilier 2011). More recent studies on SLO, in Europe, point to the broader political governance context to illustrate the relationship between acceptance and stable regulatory and legal frameworks (Lesser et al. 2021). In order to understand the importance of these contextual factors in shaping the nature of these interactions, we turn to interactive governance theory.

### Interactive Governance Theory

The interactive governance theory as used in this study consists of three components: the governing system (GS), the system-to-be-governed (SG) and the governing interactions (GI) (Jentoft and Chuenpagdee, 2015). These altogether ensure the ‘governability of the system’. According to Jentoft (2007), the governing system is social or man-made; in it, we find the institutions, steering instruments and mechanisms used in resource governance. The system-to-be governed is a combination of two entities, that is, partly natural and partly social, consisting of an ecosystem and the resources that are found in it. Governance is particularly useful in offering tools for evaluating governing interactions, that is, interactions between the systems that are being governed and the governing system. Using a tool to assess interaction within the governance framework is critical for our analysis as the nature of these relationships are central to understanding the path to SLO.

Kooiman (2008: 173) argues that the governors, the governed and the interactions between them, all contribute to the governability of the system, as do external influences. Kooiman (2008) perceive governance interactions from the actor perspective and examine concepts like participatory, collaborative and policy or management interactions. Jentoft and Chuenpagdee (2015) build upon this assumption and describe three governance interactions: hierarchical, self-governance, and co-governance. Hierarchical governance characterizes the interaction between a state and its citizens (a top-down style of intervention) expressed in policies and law. Self-governance describes a situation in which actors take care of themselves without any intervention from the government. Co-governance occurs when government acts as a constructive partner in the governance of a resource. It may take the form of partnerships or participation where no one actor is in control; thus, the interactions are horizontal.

### Tripartite SLO Framework

To investigate the interaction of actors within a governance arrangement, we developed a theoretical framework based on the Prno and Slocombe’s model of the state, society, and market interaction in mining activities (2012) and the interactive governance theory from Jentoft and Chuenpagdee (2015) to assess the quality of interaction.

The Prno and Slocombe model outlines the components of governing systems; the state, community, and company actors in the exploitation of mining resources. The model provides context to understand the goals, expectations, institutional arrangements and practices upheld in each of these governing systems. While their model is effective in describing the governance systems, it does not pay attention to the governing interactions that exist within the system. Unlike other resource industries, the mining industry involves diverse actors, and the interactions between the actors are important both for inclusion and influence regarding resource governance.

**Fig. 1:**
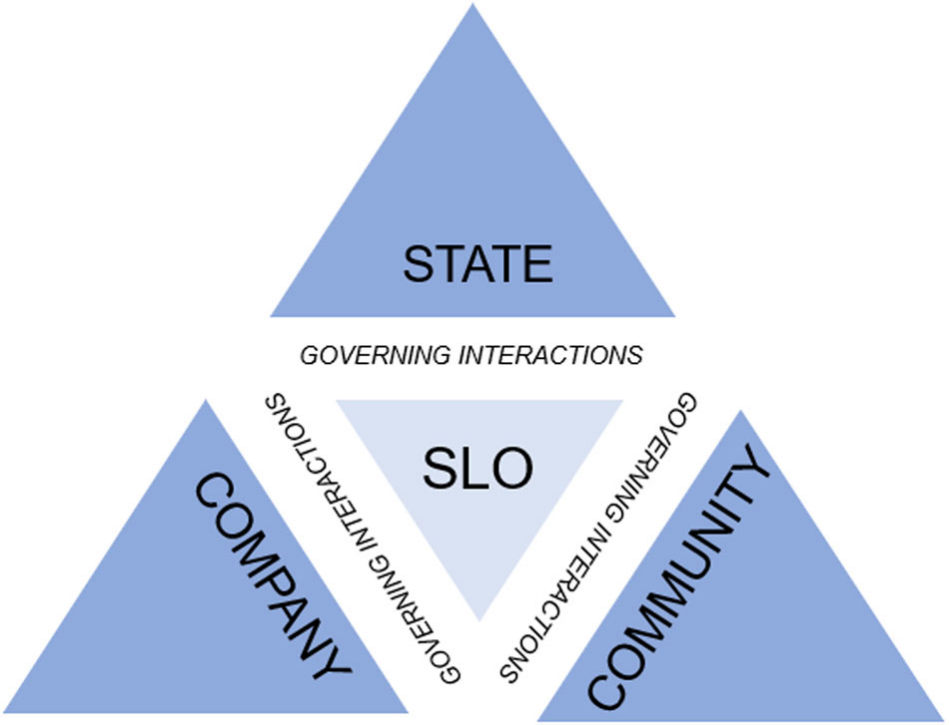
Tripartite SLO framework developed from Prno and Slocombe (2012) and Jentoft and Chuenpagdee (2015)

Coupling the Prno and Slocombe model with the Jentoft and Chuenpagdee theory allows us to study the interactions that occur in mining operations more accurately. Jentoft and Chuenpagdee first applied their theory in fishery industry which, from a resource management perspective, offers parallels to the mining sector. Combining the two models, as seen in Fig. 1 above, the Tripartite SLO Framework provides a comprehensive and robust framework that captures both the governance systems and the interactions between actors. In this study, we look specifically at the *governing interactions* to assess how the actors understand their position in relation to other actors. To test the applicability of this framework, we examine two Indigenous communities engaged with mining operations in Canada: English River First Nation, Saskatchewan, and Tlicho, Northwest Territories.

## Research Design and Methods

### Case Study Approach

The research was conducted using a comparative case study approach (Yin 2009) involving methods consistent with primary (interviews) and secondary (document reviews) qualitative methods to explore the relationship between government, industry, and Indigenous communities along with the regulatory context. This approach also allowed for an in-depth analysis of a specific subject area (Creswell 2014). To understand industry-state-community relations, we focused on the different governance regimes of two Indigenous nations/communities: English River First Nation (ERFN) and Tlicho First Nation (TNF). The cases are both located in the Canadian north – a region rich in natural resources and the homelands of Indigenous populations. Both nations/communities have relatively strong governing structures, a long history of political engagement with the federal and provincial or territorial governments on mining issues and are illustrative of relative success stories in SLO. Yet, as discussed below, they nonetheless have significant differences in their formal processes and regulatory frameworks, including Indigenous rights and governance contexts, with one (TFN) bring situated in a formal co-management regime.

#### English River First Nation (ERFN) – Cameco

The Northern Administrative District (NAD) in the province of Saskatchewan accounts for almost half of the province’s land area at 268,390 km^2^ (Statistics Canada 2012). The NAD population of roughly 37,000 lives in approximately 45 communities, which include municipalities, First Nations reserves, and settlements, sometimes in combination. The region is resource-rich and heavily dependent on industry, particularly mining. Uranium mining is the most prosperous industry in the region, accounting for 13% of worldwide uranium production in 2018 (NRCan, 2020). The two major operators in the NAD are publicly traded companies, Cameco Corporation and Orano Canada. The McArthur River mine, owned by Cameco Corporation, has been producing since 1988 (Parsons and Barsi 2001). McArthur is the world’s largest high-grade uranium operation.

Regulation of uranium mining is a federal and provincial responsibility. Federally, uranium mining and milling require licensing from the Canadian Nuclear Safety Commission (CNSC), which carries out the federal government’s legal duty to consult Indigenous peoples mainly through the federal environmental impact assessment (EIA) process (Canada: Aboriginal Affairs and Northern Development Canada 2011). The Saskatchewan provincial government also provides permits to uranium companies for exploration, undertakes its own provincial EIA process for proposed mining developments, and forges mine surface lease agreements (MSLAs) for mining proposals. Both the federal and provincial governments have developed guidelines to aid government officials and proponents in the consultation process, specifically: *Aboriginal Consultation and Accommodation* (Canada: Aboriginal Affairs and Northern Development Canada 2011) and *Proponents Guide: Consultation with First Nations and Métis in Saskatchewan Environmental Impact Assessment*. The two governments typically coordinate their EIAs, sharing consultation duties where possible, while each government must ensure that consultation meets each of their independent legal requirements.

Governance of uranium mining, namely the McArthur River mine, operates on a four-tiered system with federal, provincial, and Indigenous governance systems as well as industry, including non-regulatory co-management boards, each with different roles and responsibilities to monitor and manage the industry. ERFN, which is governed in accordance with the federal *Indian Act*, is not recognized as a government institution per se but is regarded as an ‘organization’ and does not have a federally delegated administrative role. However, despite the lack of self-government recognition, ERFN plays a prominent role in the mining development and management processes.

#### Tlicho First Nation (TFN) – Diavik Diamond Mine

The Diavik Diamond Mine is located 300 km northeast of Yellowknife, Northwest Territories (NWT), situated on a 20 km^2^ island in Lac de Gras. The NWT is home to about 40,000 people with 20,860 Indigenous people, including the Lutsel K’e Dene First Nation, the Yellowknives Dene First Nation, the Kitikmeot Inuit Association, the North Slave Metis Alliance, and the Tlicho First Nation (TFN) (Shigley et al. 2016). As a territory, the NWT does not enjoy the provincial constitutional powers set out in the Canadian constitution (Sabin 2017). However, in 2014 the federal government devolved the management of public land, water, and resources to Government of the Northwest Territories; on the basis of an agreement which was signed by federal, territorial, and nine Indigenous governments including the Tlicho First Nation. This agreement introduced new executive, fiscal and regulatory institutions to manage intergovernmental relations within the territory (Sabin 2017).

Diamonds were discovered in the NWT in 1991, leading to the establishment of Canada’s first diamond mine, Ekati – followed by the Diavik and Gahcho Kue mines. Given the novelty of diamond mining in the North and the uncertain environmental impacts, Ekati was subject to an independent review panel EIA under the *Canadian Environmental Assessment Act*. Drawing on Ekati’s experience and impact mitigation solutions, Diavik underwent a more streamlined EIA process under the federal act which, based on the mitigation plans identified by the proponent and public consultation, determined that Diavik “is unlikely to cause significant adverse effects” (Canada: The Canadian Environmental Assessment Act 1999). Diamond production at Diavik started in 2003. In 2019, the mine produced 6.7 million carats and employed 1124 people – of which 22% were northern Indigenous (Mining Data Online, 2020). Mining is expected to continue until at least 2025, but recent mining of new ore bodies may further extend the mine’s lifespan.

Diavik was proposed during a transition period in NWT governance. The EIA process began in the mid-1990s, with an impact statement report to the federal government in 1998. It was also in 1998 that the *Mackenzie Valley Resource Management Act* was established in the NWT, as part of a commitment under the Indigenous land claims agreements settled between the Government of Canada, Government of the NWT, and the Indigenous government organizations to establish a system of shared decision-making power based on co-management boards. Co-management represents an ideal in Indigenous-state collaborations (Bateyko 2003), and a “change from a system of centralized authority and top-down decisions to a system which integrates local and state-level management in arrangements of shared authority, or at least shared decision-making” (Rusnak 1997:2). A further negotiated self-government agreement between Tlicho, the Government of NWT, and the Government of Canada, established the Tlicho Government and provided the TFN with rights to pass laws enforce its own laws and regulations (Tlicho Government, 2015). The self-government agreement also contains a strong commitment to protect land and resources in the region and for TFN to participate in established co-management bodies (Gibson and Klinck 2005).

### Data Collection and Analysis

Semi-structured interviews were conducted with stakeholder representatives from each of the case studies to explore perceptions of governance challenges and opportunities. The main objective of the interviews was to gain an understanding of the governance structure and relationships relative to each mining operation. A semi-structured interview approach allowed the comparison of information provided by interviewees and the gathering of rich data through a somewhat flexible approach (Turner 2010). For document analysis, we analyzed publicly available materials for each mining operation, including EIA decision reports, annual project reports, corporate sustainability reports, newspapers, expert testimony and witness reports from project regulatory hearings, various media reports, and available academic research papers and theses based on the mines or addressing resource use and governance in each region.

All interviews were conducted one-on-one and in-person in 2019 and 2020. Due to Covid-19 restrictions resulting in no on-site access to mining operations, coupled with Indigenous governments focused primarily on responding to Covid-19 outbreaks, we concentrated our interviews on the expertise and experience of a limited number of key informants directly engaged in mining governance. A total of five key informants were engaged, with each interview session lasting between 30 and 60 minutes. The interviews were recorded with participants’ consent. Thematic analyses of interview transcripts, using Nvivo software to assess the content and identify emergent and crosscutting themes, allowed for a comparison of viewpoints (Marshall and Rossman 1999). We followed Miles and Huberman’s (1994) description of qualitative data analysis which is a cyclical process: 1) reduce data (code and organize into themes); 2) display data (quotes organized into tables that are categorized by nodes); 3) conclude (from amalgamated quotes as organized into tables); 4) verify conclusions (across data sources and through participant review of researcher interpretation and findings). The themes were analyzed to expose similarities and differences in perception, and then aligned with information from document review where applicable. Although we interviewed only a small number of participants, the individuals engaged have a long history and rich knowledge of the respective mining operations and governance systems. A primary focus of interviews was to supplement and verify document reviews and fill any gaps that document reviews were unable to bridge effectively (Creswell 2014).

## Results

Results are presented below in three parts. First, the *quality of interaction*. These are the factors central to engagement between actors in decision-making related to resource use. Second, the *outcomes of interaction*. These are the agreements and management protocols that contribute to the overall governance of the system. Third, the *relationship between actors*. This describes how participants understand the relationship between government-community, government-industry, and industry-community in shaping SLO. Table 1 lists the various institutional factors, interactions, agreements, and collaborative practices that we elaborate upon using the data from interviews and document analysis.

**Table 1:** Overview of SLO supporting factors in TFN-Diavik and ERFN-Cameco case studies

Factors	TFN-Diavik	ERFN-Cameco
Legislation and permit process	● Duty to consult ● MVRMA board comprises 50% Indigenous and 50% government representatives.	● Duty to consult ● The provincial consultation policy framework for Indigenous peoples
Indigenous rights and governance	● Constitutional rights (duty to consult) ● Signed modern treaties with the federal government ● Self-government	● Constitutional rights (duty to consult) ● Signed historical treaties with the federal government ● Saskatchewan’s First Nations and Métis Consultation Policy Framework
Industry-state agreements	● Socio-Economic Monitoring Agreement ● Environmental Agreement	● Mine Surface Lease Agreement. ● Human Resource Development Agreement
Type of interaction	● State-community: self and co-management ● State-company: hierarchical ● Company-community: privatized	● State-community: hierarchical and non-co-management ● State-company: hierarchical ● Company-community: Privatized
Industry-community agreement	● Participation Agreement	● Collaboration Agreement
Management and monitoring entities	● Diavik Environmental Agreement —Environmental Monitoring Advisory Board (EMAB) ● Communities Advisory Board	● Community Vitality Monitoring Partnership Process (CVMPP) ● Environmental Quality Committees (EQC)

### Quality of Interaction

#### Transparency and Trust

Transparency and trust emerged as a crucial element of a successful relationship between stakeholders, both for ERFN-Cameco and TFN-Diavik. In establishing transparency and trust, the cases illustrate the importance of ongoing and two-way communications about project performance and emerging issues and concerns. For both Cameco and Diavik, participants indicated that how community concerns and interests were addressed helped the companies gain the trust of Indigenous communities, in large part by the companies being transparent in what their interest was concerning the land and opening a communication channel to know and understand the interests of the affected communities. A member from ERFN recalls his experience with the mining process and how he supports the industry, noting that he had “100% faith in the whole process because I seen (sic) it all, I’ve lived through it.” Another participant emphasized the importance of transparency and honesty, explaining that “in the long run…so honesty and fairness and the ability to come to the communities is a big thing.” This was echoed by another community member, explaining that “we’re [the community] being transparent about where our revenues are coming from, we’ve been transparent on the opportunities, and we’re transparent on the mining companies that are approaching us for future work.”

In the NWT, a former senior advisor to the TFN Government was also of the view that trust and transparency are key factors to community satisfaction with the Diavik project. He said that he “recognized that it is possible to work with other stakeholders through trust and transparency” and further commented that “we are gathering strength through our own histories, finding a system that not only provides truthfulness for yourself, but a relationship built on trust with other jurisdictions that includes the feds, provinces, and territorial governments that share resources that are in your background.” In both cases, the support for mining projects has been constant through the years with limited to no disruptions or community conflict or opposition; an indication of the importance that companies are transparent with their operations and, in turn, communities have generally developed trust in the operators.

#### Acknowledgment of Indigenous Rights and Land Use

Treaty rights and traditional land rights were significant factors in the nature and quality of interactions underpinning both case studies. Project documentation and ERFN-Cameco and TFN-Diavik participants identify treaty rights and the duty to consult as foundational to the interactions that have emerged. An industry member talks about the environment and land use, for example, explaining: “we engage with communities, we create business-friendly relationships and win-win solutions to allow us to access land and mitigate any risks.” The industry participant further explains how the industry has respected Indigenous rights by signing an agreement, emphasizing that “ERFN has a traditional territory, and they’re asserting their Aboriginal and Treaty Rights that need to be respected.” Participants widely acknowledged that environmental protection in the face of mineral development was directly linked to the measures implemented by the companies in collaboration with the Indigenous governments. A community member said of the industry, “we expect them to put the land back in its original state, at least close to its original state, so there is mining companies now they put aside decommissioning funds so when the when the mining is over, there will be money for other companies to come in and clean up their whatever and put it back in its original state”. He continued by saying: “I don’t see the industry as a threat [like] I once did back then. When I first became a Chief, industry for me was a threat [and] I think industry thinks I’m a threat, but I just want what’s right…we never demand anything from anybody, we just wanted to make sure our lands are protected [so that] young people got jobs.” Another community member stated, “our first priority as a First Nation is how you are gonna (sic) protect the land to make sure that my grandchildren are still practicing their way of life…and still do mining.”

TFN rights and treaties regarding their traditional lands have also played an important role in exerting influence on mining operations, management, and the benefits received. Through engagement in co-management and by way of self-government, TFN is able to jointly manage the social, economic and environmental resources and have gained increased control over their use. Representation on co-management boards in particular has provided a stable forum for conveying observations, concerns, and grievances related to mining operations and impacts. According to the Manager of External and Internal Affairs at Diavik: “Clearly, Aboriginal groups in the Northwest Territories have won an important right to influence their participation in resource-based projects” (Hoefer 2004). This perspective was echoed more recently by Diavik’s President, indicating that the company prides itself on operating sustainably and that “the key to this is the relationships built with our Aboriginal partners” (Tlicho 2013).

#### Community Involvement

ERFN involvement in mining decision making is described by the province of Saskatchewan’s policy framework for First Nation and Metis Consultation, which enables early engagement with Indigenous groups and laying the basis for negotiation among partners. As one government official explained, the province will “push companies to meet very early in the process and have a strong engagement process.” They also noted that the province has “really strong requirements to improve on engagement”, which helps support “a lot of potential development for other minerals as well.” Participants also emphasized improvements over time in community engagement in mining operations, with a community member explaining the changes from the past to the present as follows: “When we first started like I said I know there was zero way or no way, but over the years through the courts and through protesting, through arguing with government, we finally got that consulting thing done…and under Supreme Court, it says you cannot put obstacles in front of First Nations… it gives the First Nation a little more control over environmental issues”. Discussions with interview participants emphasize that there remains room for further improvement, but that engagement with the community is seen as positive overall and has continued to improve over time.

TFN has taken a slightly different route, choosing to engage more directly with industry and government. Through Indigenous self-government, TFN has taken decision-making into their own hands, establishing their own rules and laws, and making allies in environmental management for mineral development. Grand Chief Edward Erasmus, for example, acknowledged the significance of traditional knowledge in his closing comments at the public hearings for the Diavik project, commenting: “yesterday we made history for the first time, traditional knowledge was recognized and is being considered in this process, and we would like to thank the [Review] Board for that” (2012). These comments reflect the value placed on long-term relationships by both the industry and the community and reinforce the extent to which Indigenous communities are to be meaningfully incorporated into the decision-making process. Responses from participants, coupled with statements made by communities in the public hearing process for project approval and via the more recent participation agreement and community advisory board, suggest that Indigenous communities have gained an official voice in managing resource development projects located in their traditional territory.

### Outcomes of Interaction

Project documents and interviewees indicate that stakeholders were involved in many aspects of the mine process, from the initial environmental assessment to community development initiatives and monitoring performance via committee boards. These collaborative practices enabled transparency and engagement and have since become preferred methods for industry to communicate with Indigenous communities and ensure that a project’s SLO is enduring. In parallel, Indigenous communities have sought additional ways to mitigate the potential socio-economic and biophysical risks of mining projects. For both ERFN-Cameco and TFN-Diavik, negotiated agreements have played a significant role in relationship-building between project proponents and Indigenous communities.

In the ERFN-Cameco case, a collaboration agreement was signed to ensure that the mining operation would continue to deliver economic benefits to ERFN communities; the agreement also established an ERFN-led committee to oversee industry’s activities. As one community member stated, “I’m very confident and very supportive because we need jobs for young people…they are sort of spindles from the industry [and] we get all the spindles to contract work here - that’s how we build our industry.” Participants revealed in their comments that the mining industry provides important economic benefits through local jobs and income generation, and that these benefits are important for the communities and in stabilizing ongoing community-industry relationships. An industry participant similarly emphasized the importance of the collaboration agreements, explaining that “Cameco signed a confidential collaboration agreement with its primary communities, the agreement builds on the historic relationship as well as a commercial relationship with businesses owned by the communities”. The participant went on to explain that the collaboration agreement is a product of respect, and a direct effect of Aboriginal and Treaty Rights, noting that “we’ve chosen to enter into these agreements, and they get direct compensation from us.”

TFN-Diavik are engaged in three agreements, namely a socio-economic agreement, an environment agreement, and a confidential participation agreement. When the mine was approved, a socio-economic agreement was signed between Diavik, the Government of the NWT, and the Tlicho Government – in addition to other Indigenous groups. The agreement, required as a condition of project approval, formalizes local employment, training, and capacity building commitments. An environmental agreement was negotiated detailing Diavik’s commitments to impact management, and a community advisory board formed for monitoring those commitments and setting out a formal mechanism for consultation and dispute resolution. The participation agreement, signed between TFN and Diavik, was a confidential agreement detailing further company commitments on matters such as local contracts, revenue, and business ventures. Tlicho Grand Chief Eddie Erasmus stated during the renewal of the participation agreement with Diavik: “The Tlicho Government is pleased with the outcome of this updated agreement and the continued benefits that will be realized by the Tlicho people for the remainder of the Diavik Diamond Mine,” and that “this renewed agreement is a reflection of the strength of our relationship with Diavik.” In support of this, the Diavik’s Chief Operating Officer noted that the agreement “reflects our continued commitment to ensuring that we do all we can to ensure that the Tlicho people realize meaningful benefits, including continued training, employment, and business benefits.” Results highlight the importance of industry engagement in community-company interactions, activities, and partnerships external to, or in addition to, formal regulatory processes. Missens et al. (2007) characterized Diavik as an example of “thinking beyond extraction” and “supporting communities and their enterprises.”

### Relationship Between Actors

All participants noted the importance of government, industry, and Indigenous communities having a clear and common understanding of roles and the legal consultation obligations of government. The governing interactions involve forms of communication, relationship, and participation between and among governing systems.

#### Government-Community

The ERFN is a band as defined in the *Indian Act*. As such, ERFN operates within the confines of that Act, making decisions through a band council, which is responsible for managing the day-to-day affairs of the band, but which are ultimately a federal responsibility. The governing interactions are hierarchical. Legally, ERFN is an organization and not a government per se and therefore does not have any delegated authority from the Crown; however, ERFN is consulted and invited to participate in public hearings for mining developments and in other participatory licensing and permitting processes. Even though ERFN does not enjoy the autonomy of Indigenous self-government, a member of ERFN expressed that the current regulatory process is “handled very well,” adding that for the protection of resources Indigenous people can rely on their established rights. The participant explained that engagement and influence is handled according to the licensing process for development and that “it’s regulated in a better and honest way, not a forceful way…. a very respectful and honourable way of enforcing the policy guidelines.” The individual went on to explain that “it seems to be working…I’m not saying it’s all perfect, but better than 20 years ago, hundred per cent.” When asked about their interactions with the ERFN community, a government representative suggested that there is “not a lot of direct contact with the First Nation” but rather much of the engagement is by way of the established environmental quality committee for the mine, which includes representatives from both stakeholders to share information about uranium mining, its impacts, and management solutions. The participant said that government “has a rep on this committee, and the committee visits uranium mines, [and is] involved in monitoring at the existing mines.” They further emphasizing the importance of participation of the community in the management of mining and maintaining government-community interactions.

The TFN-Government interactions have created a unique and evolving government-to-government relationship with the federal and territorial governments. TFN is no longer a band under the *Indian Act*, with autonomy secured after signing of the Tlicho agreement. As one participant explained, “with self-government, the *Indian Act* is not bound to us” the TFN has the ability to manage natural resources extraction on their traditional lands. In 2012, the Tlicho Government and the territorial government signed a memorandum of understanding titled ‘Working Together’ – a formal recognition of a government-to-government relationship. As described by Everts-Lind (Tlicho Government 2013), this relationship is understood as a partnership; a comment echoed by a TFN representative who noted the positive change in interactions owing to a shift in governance. The Diavik approval process, and subsequent participation agreements between TFN and Diavik, were perceived as significant achievements by the Tlicho, empowering the TFN with the ability to make their own decisions and determine their own futures.

#### Government-Industry

The governing interactions between government-industry in both cases follow a hierarchical pattern, depicting a typical top-down channel of communication grounded in regulatory processes. In the case of Cameco, for example, when asked about interaction with state actors an industry participant said that for uranium mining in particular the operations “are subject to strict [federal] regulations, every aspect is subject to licensing – exploration, site preparation or construction, operating, decommissioning and release from licensing.” A provincial government representative simply echoed the hierarchical approach grounded in regulation at the federal level, and went on to explain that currently the provincial government “is looking at current involvement in that process” including in mineral exploration discovery, and “whether government should have a more active role in speaking about expectations”. Another government representative confirmed that the government is “involved in delivering permits which includes the lease and exploration” and that decision making on crown land is at the “behest of the province”. She further prioritized engagement with the community and suggested that “duty to consult is central”, and the best way for the industry to engage and communicate is to follow the consultation policy framework established by the province. She concludes by saying that “the contacting process is very structured and outlined clearly in policy, assessment on minor and major impact”.

In the Diavik case, the NWT, as an independent entity, makes all rules and regulations guiding natural resources, including diamond mining in the territory. Decision-making authority for natural resources rests primarily with territorial and Indigenous governments, with much less interaction with the federal government.

#### Industry-Community

Notwithstanding regulatory processes, many of the responsibilities for managing the impacts and benefits of mining, and maintaining relationships, rests with the industry and the Indigenous communities, which has led to a win-win outcome and a SLO. Participants for both case studies, from industry and community, explained that they are mostly involved in major decisions with no interference from the government. An industry member touched on the fact that there is a strong and enduring relationship with communities based on history. She stated that the cooperation agreement in place “builds on the historic relationship as well as commercial relationship with businesses owned by the communities.” The participant went on to explain that engagement between Cameco and the communities occurs primarily through an established process (i.e. community and industry representatives) to meet regularly and discuss operational and environment-related matters of importance to the communities. Similarly, an industry participant commenting on the Diavik project notes that: “Our participation agreements, which we have with the Tlicho Government and four other Aboriginal communities, are all based on mutual respect, active partnership, and long-term sustainable commitments” (Tlicho 2013).

Both Cameco and Diavik have been lauded for best practices in Canada regarding Indigenous relations and recognized as leaders in both social innovation and sustainable development. The Mining Association of Canada, for example, characterizes Cameco as “a global leader in Corporate Social Responsibility” and has “been honoured for progressive Aboriginal relations three times by the Canadian Council for Aboriginal Business in recognition of its commitment to recruit, retain and advance First Nations employees within the organization”. Diavik has also been recognized as a “four-time Canada’s Top Employer in 2011 as a leader in Aboriginal relations and was re-certified with the prestigious Gold level of achievement under the Canadian Council for Aboriginal Business’s Progressive Aboriginal Relations program” (Natural Resources Canada 2014).

## Discussion and Conclusion

Assessing the interaction at mining projects that enjoy SLO illuminates several important factors related to governance. Using the Tripartite SLO Framework allowed us to make the connection between the interaction of actors to the role each holds in the governance structure. While many of the relational qualities confirm previous SLO research, the relationship between government, industry, and community can be re-examined.

First, our cases underscore the importance of community involvement and recognition of rights in facilitating high levels of trust in the community to company relationship. This confirms a study by Moffat et al. (2016), which demonstrated how the roles of trust, fairness and governance may underpin the development of sustainable relationships between industry and community. Further, our analysis shows that the role of governance and legal rights of Indigenous communities made it easier for industries to work with Indigenous communities by involving them in the governing interactions to prevent conflicts. Indigenous rights shape the relationships between stakeholders and how those relationships, and especially negotiated agreements between Indigenous communities and mining proponents, influence policy and law-making regarding Indigenous peoples’ encounters with mining (Le Meur et al. 2013). Critically, having these rights and autonomy does not stifle mining development, it seems to facilitate it. This contrasts with some literature that argues local Indigenous rights will undermine resource development. Other studies argue that stakeholders believe that the legal rights of Indigenous groups will give them the power to prevent industries from operating on their land or delay mining development, thereby categorizing Indigenous groups as anti-mining. Research conducted by Lawrence and Mortirz (2019) makes this argument, where responses from Swedish mining participants indicate that rights such as free prior and informed consent are viewed as a ‘carte blanche veto’ for Indigenous people. Another study by Howitt (2001) revealed how resource-based industries in Australia made the either-or argument that any attempt to legislate for Indigenous rights threatened the economic viability of the mining industry and, consequently, the national interest.

Second, both cases studies suggest that with a reduced role for government, industry-community relationships are more active and result in better outcomes. Our findings show that in both cases, government remains a passive actor in the governance structure and the activities of industry and community are dominant, making their relationship key. Previously, the role of the government is seen to balance the economic benefits of projects and managing the expectations of all stakeholders concerned (Falck et al. 2015). But even now, this arrangement is shifting to a completely industry-community relationship where the government plays little role in the SLO process. For example, both Cameco and Diavik have paid considerable attention to their relationship with Indigenous communities and engaged them throughout the whole mining process. As we point out in our analysis, the agreements and management boards that govern the economic, social, and environmental outcomes of the project are made primarily between the community and industry. These findings are consistent with other studies (Cameron and Levitan 2014; Harvey and Brereton 2005, Harvey and Bice, 2014; Prno and Slocombe 2012; and Kemp 2010) which argued that a shift in the roles and responsibilities of government now sit with industry and community in decision-making. In other words, the community and industry decide whether they need the government in their engagement and what exact roles the government can play. Scholars have explored the benefit of government absence during the industry-community relationship as a useful tool for local Indigenous communities to leverage their rights and interests in a way that is more effective than doing so with the government (Caine and Krogman 2010; Cameron and Levitan 2014).

Third, Indigenous communities do not fit within the existing definition of ‘community’ in SLO governance models. Our findings argue that the community is not just residents or interest groups, but an authority with tremendous power to decide issues concerning its natural resources. In both case studies, participants spoke in a way that reflected their community’s autonomy and, in turn, the expectations they had of government and industry to respect that autonomy. However, this is not necessarily how ‘community’ is defined or understood in much of the governance and SLO literature. Community is commonly viewed as part of a civil society that shares common faith, culture, and ideology (Walzer, 2003), including more dispersed ‘communities of interest’ that may be globally distributed (Cullen-Knox et al. 2016; Moffat et al. 2016) or are categorized as a “network of stakeholders usually with political differences of opinions within the network of stakeholders” (Boutilier & Thomson 2011, p. 3). In the ERFN-Cameco and TFN-Diavik contexts, the ‘community’ is a form of authority in terms of resource governance and capacity that is distinct from the state. Ignoring the autonomy of Indigenous governments or failing to recognize them as such may lead to failure of any project in Canada (Papillon and Rodon 2017). This finding aligns with other researchers who believe that recognizing Indigenous communities as autonomous entities promotes respect because it elevates their standing as a government, rather than depreciating their role in decision-making to the status of a social group (Papillon and Rodon 2017).

In conclusion, our findings provide insight to the conceptualization of SLO and important practical implications for resource development. The quality of interaction between actors in our cases echoes much of the previous work on SLO and the emphasis on building trust via engagement in decision making and partnerships (Boutilier & Thomson 2011; Bursey and Whiting, 2015; Dare et al. 2014). However, the cases illustrate the importance of reinterpreting the alignment of actors, particularly the role of ‘community’ or ‘civil society’ in the governance model (Prno and Slocombe 2012). In this case, the Indigenous communities, ERFN and TFN, assume both roles of ‘government’ and ‘community’, working towards desirable socio-economic and environmental outcomes via the shared management of the mining operations. However, these cases represent conditions conducive for collaboration at the community level. In Canada, the impetus for dialogue spurred by Duty to Consult and comfortability with devolution of power within a federal system provide the contextual conditions for Indigenous communities to take a lead governance role. An opportunity for future research with this analytical framework is to assess whether the potential exists for Indigenous communities in unitary states to assume a similar role. Nevertheless, this type of governance arrangement provides a blueprint for SLO in resource development, allowing communities to partake in resource management, both in the mining sector and beyond.
